# The Integration of Optical Stimulation in a Mechanically Dynamic Cell Culture Substrate

**DOI:** 10.3389/fbioe.2022.934756

**Published:** 2022-07-19

**Authors:** Matthias Imboden, Sophia Chen, Olexandr Gudozhnik, Corey Pollock, Josh Javor, David Bishop, Herbert Shea, Samuel Rosset

**Affiliations:** ^1^ Soft Transducers Laboratory, École Polytechnique Fédérale de Lausanne (EPFL), Neuchâtel, Switzerland; ^2^ Biomimetics Laboratory, Auckland Bioengineering Institute, The University of Auckland, Auckland, New Zealand; ^3^ Department of Mechanical Engineering, Boston University, Boston, MA, United States

**Keywords:** mechanical stimulation, optical stimulation, soft actuator, dielectic elastomer actuator, microoptoelectromechanical system, mechanotransduction, optogenetics

## Abstract

A cell culture well with integrated mechanical and optical stimulation is presented. This is achieved by combining dielectric elastomer soft actuators, also known as artificial muscles, and a varifocal micro-electromechanical mirror that couples light from an optical fiber and focuses it onto the transparent cell substrate. The device enables unprecedented control of *in vitro* cell cultures by allowing the experimenter to tune and synchronize mechanical and optical stimuli, thereby enabling new experimental assays in optogenetics, fluorescent microscopy, or laser stimulation that include dynamic mechanical strain as a controlled input parameter.

## 1 Introduction

We present a multifunctional platform that combines the fields of mechanotransduction and optogenetics. It enables the application of mechanical strain to a 2D cell culture using a bioinspired electroactive polymer soft actuator embedded in the cell culture membrane while simultaneously submitting the culture to a localized light beam with tunable size, intensity, and controllable position. We have characterized the performance of the platform, demonstrated how the optical beam steering can be synchronized with the mechanical stimulation, and have produced a novel tool, which is ready to be used for future biological experiments combining light and mechanical stimulation.

Mechanobiology investigates the effect of mechanical loading on cells and tissue: the modification of their mechanical properties, their molecular response to applied stress and strain, such as stretch-activated ion channels, or the modulation of enzyme activity (Ingber, 2006; Jaalouk and Lammerding, 2009). The dysfunction of the mechanotransduction pathways has been linked with many lethal or chronic diseases, such as cancer, heart failure, atherosclerosis, arthritis, and osteoporosis (Ingber, 2003; Jaalouk and Lammerding, 2009). Bioreactors that can submit a cell or tissue culture to mechanical strain enable the reproduction of *in vitro* dynamic mechanical loadings that are representative of what cells are experiencing *in vivo*. For example, (Kim et al., 2012) designed a “gut-on-a-chip” consisting of a soft PDMS microfluidic chip which can submit a culture of human intestinal epithelial cells to fluid flow and dynamic cyclic strain. They showed that cells cultured on the dynamic organ-on-a-chip developed folds similar to intestinal villi’s *in vivo* structure, while cells grown on standard rigid culture plates did not. Other examples of mechanotransduction studies include the effect of stretch on cancer cell migration (Kamble et al., 2017) or the *in vitro* models of traumatic brain injury (Wu et al., 2021). Submitting cells to mechanical loading is usually performed by culturing cells on a stretchable membrane that is deformed by different actuation principles, such as pneumatic actuation (e.g., the commercial Flexcell^®^ Tension Systems^1^), electric motor (e.g., the commercial automated cell stretcher from Strex^2^), or more recently dielectric elastomer actuators (DEAs).

DEAs are rubbery capacitors consisting of a soft elastomeric membrane sandwiched between two stretchable electrodes (Pelrine et al., 2000). When a voltage is applied between the electrodes, the electrostatic force created by the electrical charges induces a local deformation of the membrane. The actuators can be embedded into the culture membrane, leading to very compact devices that can fit under a microscope and induce in-plane deformation, thus making live observation of the stretching process possible (Poulin et al., 2016; Rosset et al., 2017). DEAs can induce large strain amplitude at high speed thus recreating physiological strain rates or surpassing them (Imboden et al., 2019). Finally, by changing the design of the actuator, such as the arrangement of the electrodes, DEAs can be used to generate uniaxial tensile strain (Poulin et al., 2016; Imboden et al., 2019), biaxial tensile strain (Costa et al., 2020), biaxial compressive strain (Cei et al., 2016), or a combination of them (Poulin et al., 2018a). DEAs have been applied to the mechanical loading of cells in a range of applications, including change of morphology of lymphatic endothelial cells (Poulin et al., 2016) or fibroblasts (Costa et al., 2020) submitted to cyclic stretching or the study of impulse propagation in a strand of cardiac tissue submitted to dynamic strain (Imboden et al., 2019). Due to their high strain capabilities, fast response speed, and possibility to generate different strain orientations, DEAs are a versatile solution for the mechanical loading of cells. They make it possible to reproduce physiologically relevant strain profile and to submit an *in vitro* cell culture to mechanical stimuli mimicking *in vivo* conditions.

Optogenetics is a relatively new field of cellular biology that uses light-sensitive proteins for targeted stimulation or silencing of active cells (Scanziani and Häusser, 2009; Deisseroth, 2010; Ferenczi et al., 2019). It was made possible thanks to the work of (Boyden et al., 2005) who showed that a microbial opsin, a light-sensitive ion-transporting membrane protein, could be expressed in neurons, thus enabling the control of their electrical activity with light. The combination of optical and genetic methods enables one to address cells with high specificity. It has many applications, which led to its selection as the method of the year in 2010 by Nature Methods (2010). Initially, this approach was used to control the cell membrane potential of neurons, where light can be used to target individual cells selectively and initiate (or prevent) an action potential, thus controlling muscles or even behavior (Deisseroth, 2010). Optogenetics can be used as a diagnostic tool, such as for the study of cerebral blood flow, with light-induced triggering of vessel constriction to assess the contractile properties of individual cell types (Hill et al., 2015). Optical stimulation of brain cells has also been used as a therapeutic method, such as reducing pericyte-derived scarring (Dias et al., 2018) or promoting functional recovery after a stroke (Cheng et al., 2014). Optogenetics is also applied to heart muscles with the production of optically excitable heart tissue (Jia et al., 2011; Entcheva, 2013). One of the key advantages of optogenetics is the high spatial resolution provided by light illumination in comparison, for example, to chemical activation, thus enabling or inhibiting pathways on selected groups of cells. The required wavelength for optogenetic stimulation depends on the targeted opsins and ranges from 470 to 630 nm, with light intensities of a few mW mm^−2^, and pulses of 5–10 ms (Boyden et al., 2005; Lin et al., 2013).

**FIGURE 1:**
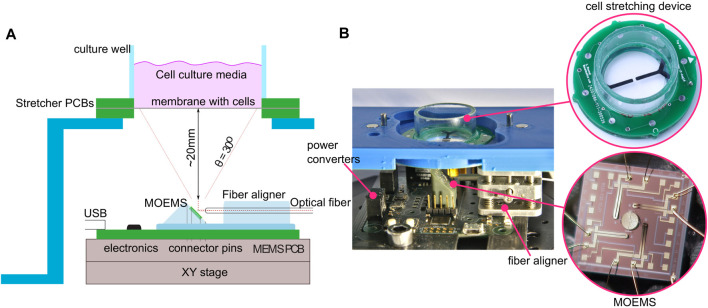
Illustration of the platform combining mechanical and optical stimulation. **(A)** Diagram of the MOEMS mounted on a PCB control board at a 45° angle. Light coming from a bare optical fiber is then reflected off the micro-mirror, through the transparent membrane, and onto the cells. The micro-mirror can be tilted along two axes to focus the spot at different locations of the culture. The platform is placed on an x-y stage to adjust the neutral spot position with respect to the cell culture. **(B)** Close-up view of the prototype. A laser diode is integrated directly to the board; the optical fiber is guided to the MOEMS with an onboard miniature x, y, and z positioner.

Here, we combine the ability to apply mechanical loading to cells with targeted light-induced stimulation in a compact platform that enables researchers to conduct experiments at the crossroad of mechanotransduction and optogenetics (Figure 1). The stretching device enables researchers to recreate *in vitro* the mechanical stimulation experienced by cells *in vivo*, thus giving the benefits of the optogenetics toolbox on mechanically strained cultures. The stretching of the cells is provided by dielectric elastomer actuators. The targeting and focusing of the illumination at precise locations of the culture is performed by a microoptoelectromechanical system (MOEMS) in the form of a gold-coated micro-mirror suspended by four thermal actuators (Morrison et al., 2015). A previous work has demonstrated resonant actuation (Morrison et al., 2017) of such micro-mirrors and how light is coupled with an optical fiber (Pollock et al., 2017).

This work demonstrates how the DEA cell actuator and the MOEMS can be integrated into a stand-alone system for synchronized high-speed mechanical and optical probing of cell tissue. One example application includes studying the effect of high strain rates on the propagation velocity of the action potential in cardiac tissue. In a previous study, Imboden et al. have used a DEA to strain a strand of cardiomyocytes at a high strain rate. A stimulation electrode induced an action potential at one end of the strand, while recording electrodes along the strand were used to record its propagation (Imboden et al., 2019). Replacing electrical stimulation with optical stimulation would enable the triggering of an action potential at any location along the strand, including in the stretched area where the dynamic strain is applied, which a fixed stimulation electrode does not allow.

In a different context, the platform can be used to identify the genes and communication pathways that influence the scar formation process in the brain in case of traumatic brain injury (Wu et al., 2022). By illuminating transfected brain cells located on the strained and non-strained (i.e., control) regions of the cell-stretcher and at different time points during the scar formation process, it becomes possible to study the influence of particular genes in the scar formation process.

## 2 Device Architecture

### 2.1 Summary of the Implemented Technologies 1: DEAs for Cell Stretching

**FIGURE 2:**
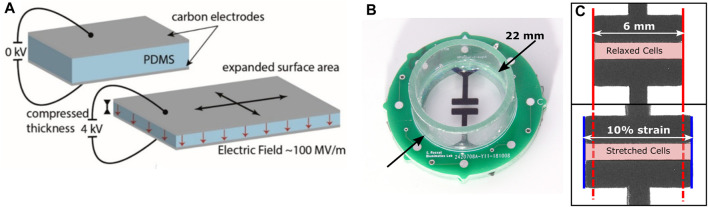
**(A)** Functioning principle of a DEA: When a voltage is applied between the electrodes, the electrostatic pressure squeezes the membrane, leading to a surface expansion of the stretchable electrodes. **(B)** Picture of a DEA-based cell stretcher. **(C)** Close-up of the central region of the stretcher. When a voltage is applied (bottom), the electrodes expand in the horizontal direction by 10%. Cells located within the highlighted area are stretched upon application of the voltage.

The cell culture well is built around a silicone membrane which serves as a mechanically active cell culture substrate. The mechanical stimulus is based on a previously described DEA actuator (Poulin et al., 2016). This enables the generation of linear, uniform strain at the center of the culture well up to an amplitude of ∼15 %. The strain is imposed by applying a voltage (typically up to 4 kV) between two carbon-loaded electrodes, which induces a Maxwell stress in the elastomeric membrane, causing it to reduce in thickness and expand in area (Figure 2A). The cell stretcher consists of a stretched membrane clamped between two printed circuit boards (PCBs) that provide electrical connection between the external power supply and the stretchable electrodes. A polycarbonate cylinder glued on the top PCB acts as a culture well (Figure 2B). The choice of the electrode geometry, along with the non-equi-biaxial pre-stretch, results in a central region that stretches uniformly and uniaxially when a voltage is applied between the electrodes (Figure 2C). This region is transparent, thus enabling optical studies of the strained cells. Cells can be selectively cultured in the stretched region between the two electrodes or over the complete device. In this latter scenario, the cells located at the periphery of the device remain unstrained and can serve as control. Beyond the ability to apply a strain to an *in vitro* cell culture, the DEAs can do so extremely fast (Poulin et al., 2018a). Typical strain rates can reach 3% ms^−1^, and well exceeding standard physiological strain rates observed. Furthermore, by applying feed-forward overdrive signals (Poulin and Rosset, 2019), the DEA can reach 10% strain in well under 1 ms, generating effective strain rates far exceeding 100 s^−1^, corresponding to rates associated with impact and result in trauma (for *in vivo* case).

The fabrication of the DEAs used in this study is based on a process similar to Poulin et al. (2018b) and Wu et al. (2022). The devices can be sterilized in an autoclave without affecting the actuation properties (Wu et al., 2022).

### 2.2 Summary of the Implemented Technologies 2: MOEMS

**FIGURE 3:**
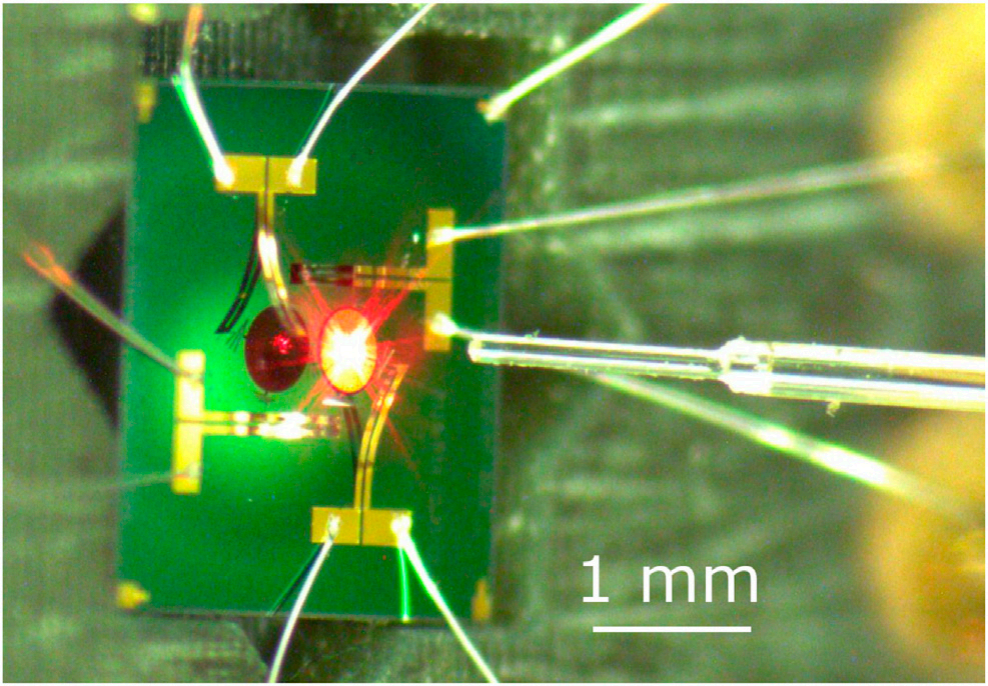
Example of a varifocal MEMS device. The silicon chip is 2.5 mm across. The central micro-mirror has a 400 µm diameter and can optically deflect light over a ±30° range along two axes. The focal point can be dynamically tuned from −10 mm to infinity. The optical fiber is centered ∼1 mm from the micro-mirror using a custom two axis fiber positioner.

The light is coupled from an optical fiber to the cell culture *via* an electrothermally actuated micro-mirror with a diameter of 400 µm (Figure 3). Beyond steering the reflected light ±30° along two axes, the micro-mirror is curved and can be heated to adjust the focal length. Like this, divergent light from an optical fiber can be steered and focused to the desired position. Full details on the MOEMS design and performance has been reported previously (Morrison et al., 2015). The focal point can be tuned from −10 mm to infinity at a timescale of 15 ms, and the micro-mirror can achieve vibration-free positioning within 2 ms. The MOEMS was initially designed for indoor communication and the coupling to an optical fiber has also been previously implemented (Pollock et al., 2017). There it has been shown that the divergence of 1.5 micron light can be limited to 0.18°, resulting in a spot size (defined by the FHWM) of 0.6 mm at a distance of 100 mm from the optical fiber. The optimal mirror-fiber separation is defined by the optical opening angle of the light emitted from the fiber and the micro-mirror size. It is crucial to contain the light within the micro-mirror to avoid stray light from illuminating cells that are intended to be left in the dark.

### 2.3 Technology Integration

**FIGURE 4:**
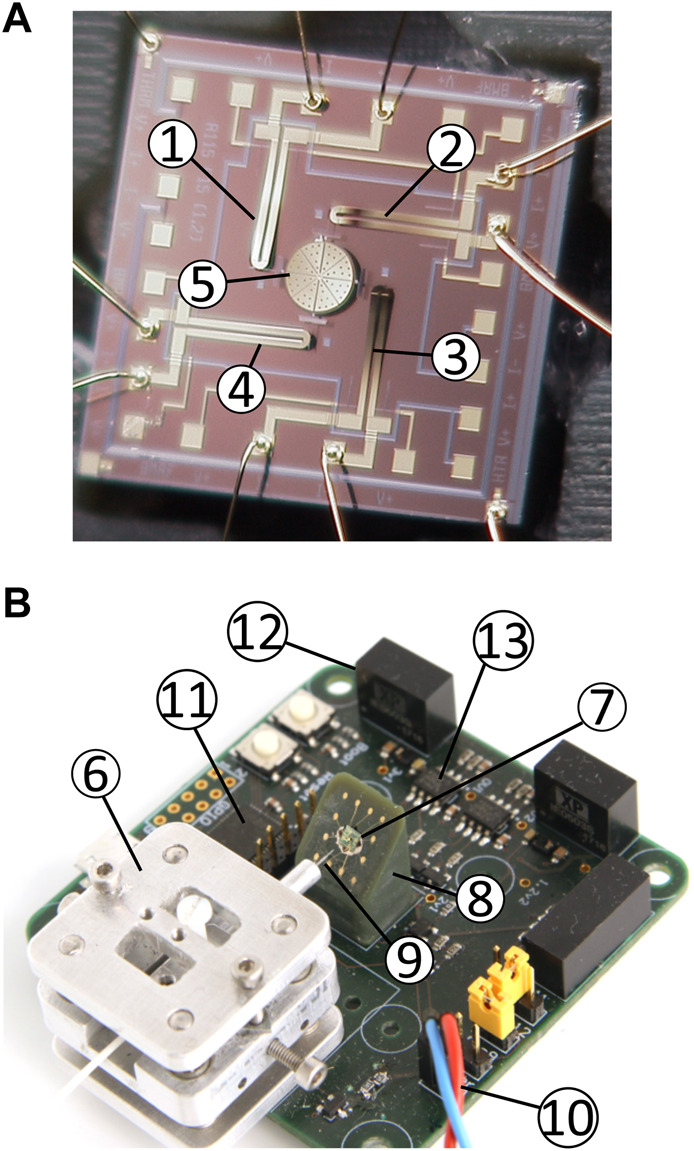
Components of the platform. **(A)** MOEMS chip (2.5 × 2.5 mm^2^). ➀-➃ bimorph 1 to 4 and ➄ curved micro-mirror. **(B)** Picture of the assembled PCB. ➅ x-y-z fiber aligner, ➆ micro-mirror, ➇ 45° tilted holder, and ➈ 4.3 μm/125 µm optical fiber ➉ electrical connection to the laser diode ⑪ STM32 microcontroller ⑫ DC/DC converters for the thermal actuators ⑬ CMOS digital isolators.

The micro-mirror chip (Figure 4A) is glued on a custom-made 3D printed holder with contact pins. The surface on which the chip is glued is tilted by 45°. The chip pads are then wire-bonded to the contact pins of the holder.

The holder can be press-fitted on a custom PCB used to control the platform (see Supplementary Material for more details on the control circuit). For ease of implementation with a digital microcontroller (STM32L433), the heating power applied to the thermal actuators is controlled using Pulse Width Modulation (PWM). The microcontroller is driven by a 80 MHz clock, and the frequency of the PWM signal is set to 10 kHz, thus providing a duty cycle precision of 0.0125%. The period of the PWM signal is about 1 order and 2 orders of magnitude shorter than the thermomechanical response time of the tilting bimorphs and of the micro-mirror focus, respectively. The thermal time constant of the actuators therefore acts as a low-pass filter, and the PWM signal effectively behaves as an averaged analogue signal. The bimorphs are controlled with 1.2 V (at 100% PWM), and the micro-mirror focus with 24 V. DC/DC converters are used to generate the required voltage from the 5 V input provided by the USB bus. The voltage applied to the bimorph is floating, which enables to set the bimorphs at different relative potentials. Bimorphs 1 and 2 (c.f. Figure 4A) share a common reference, and so do bimorphs 3 and 4. Adjusting the potential difference between the two groups controls the amplitude of the current flow through the micro-mirror base, and hence the curvature of the micro-mirror. A PWM signal is also used to drive the laser diode (Qphotonics QFLD-635-1SAX, 635 nm, 1 mW), thus allowing to control its intensity. The USB bus provides a serial interface to send commands from a computer to the platform. Assuming that the micro-mirror focus and laser diode power are kept constant, rapid motion of the micro-mirror requires sending four commands (one per bimorph). All commands are 8 bytes long sent at 115,200 Bd, thus enabling to change the micro-mirror position 450 times per second.

A custom-made fiber aligner is mounted on the PCB and provides precise positioning of the fiber along the three orthogonal axes. The fiber can be slid through a cannula to adjust the distance between its extremity and the micro-mirror. Screws with counteracting springs enable the shifting of the cannula up/down and left/right. These two degrees of freedom position the fiber so that the laser beam hits the center of the micro-mirror (such as shown in Figure 3).

Figure 4B shows the PCB with all components required to control the optical stimulation side of the platform. A total of four holes in the PCB are designed to install spacers to position the DEA cell stretcher above the micro-mirror. However, for this testing phase, we have decoupled the platform’s optical and mechanical stimulation parts. The mirror-control PCB is placed on an *x* − *y* − *θ* stage, while the cell stretcher is positioned on a fixed platform (c.f. Figure 1A). This configuration makes it possible to manually adjust the laser spot’s impact position on the cell stretching device.

## 3 Device Performance and Characterization

### 3.1 Controlling the Mirror and Tracking the Spot

To steer the light over the membrane, we use antagonistic actuation. First, we define the maximal power *PWM*_max_ to apply to the bimorphs. High applied electrical power can lead to permanent deformation of the thermal actuators or their destruction in the worst-case scenario. Consequently, it is safer to choose a conservative maximal power at the cost of a reduced tilt angle range. As the micro-mirrors are experimental devices, their performance and parameters (such as the electrical resistance of the bimorphs) vary from batch to batch, and the value for the maximal power must be defined for each of them starting from a conservative value and then incremented. In terms of the PWM setting applied to the bimorphs, we have used maximal power (i.e., maximal duty cycle values) in the range of 10–30% of the PWM signal period. Once the value of *PWM*_max_ is chosen, the platform is initialized by applying a baseline power of *PWM*_max_/2 to all bimorphs, which effectively produces a piston effect pushing the micro-mirror toward the substrate and represents the neutral position of the spot. The mirror setpoint is defined in a normalized coordinate system in which each axis (x and y) can be set in a range of −1 to +1, with (0,0) representing the neutral position. The pair of bimorphs controlling each axis (c.f. Figure 4A) is operated antagonistically. For example, to move the spot in the positive *x* direction the power of bimorph 3 is increased, while the power of bimorph 1 is decreased by the same amount. The relation between a normalized setpoint point (*x*, *y*) and the PWM signal applied to each bimorph is given as follows.

$$PWM_{1}=\left(1-x\right)\frac{PWM_{\max}}{2},$$ (1)

$$PWM_{2}=\left(1+y\right)\frac{PWM_{\max}}{2},$$ (2)

$$PWM_{3}=\left(1+x\right)\frac{PWM_{\max}}{2},$$ (3)

$$PWM_{4}=\left(1-y\right)\frac{PWM_{\max}}{2},$$ (4)

with index 1 to 4 representing the bimorphs, as numbered in Figure 4A. The relation between the mirror setpoint (*x*, *y*) and the effective laser spot position (*X*, *Y*) on the membrane in real-world coordinates depends on many parameters including membrane-mirror distance or the relation between the PWM value and the curvature of the bimorphs (which can vary between micro-mirrors). The power applied to the micro-mirror for the control of the focal length, and for the intensity of the laser diode can be set between 0 and 100% without risk of damage.

A LabVIEW interface controls the micro-mirror and acquires images from a camera (IDS UI-3880LE-C-HQ) with a zoom lens (Computar MLM-3XMP) mounted above the cell culture. Once the magnification and focus of the lens have been set for an experiment, a picture of an array of dots with 5 mm spacing is taken and fed to the LabVIEW image processing toolbox to correct for lens distortions and to convert pixel coordinates into real-world units. Image processing is used to locate the position of the laser spot using the red channel of each RGB frame. First, a low-pass filter is applied to reduce the speckle contrast produced by the screen, followed by an automatic threshold using the moment method. Finally, a particle analysis routine is applied to locate and characterize the spot (coordinates, area, semi-axis length, etc.). The size of the spot depends on the camera parameters (exposure, gain, and brightness) and does not represent the FWHM value. In the next section, we defined targeted displacements in the normalized coordinate plane and measured the resulting laser spot trajectory in real world coordinates using the camera and image processing routines.

### 3.2 Steering the Light Over the Membrane

A holder with a micro-mirror was inserted on the PCB. The fiber was then aligned to center the laser spot on the micro-mirror. Because silicone membranes are transparent, a translucent paper screen was used as a mock-up of the cell stretcher membrane to characterize the steering ability of the micro-mirror, as this enabled to image the laser spot with the camera. The screen was placed 19 mm above the micro-mirror. The maximal power applied to the bimorphs was set to *PWM*_max_ = 30% to avoid overheating the actuators. Different shapes (circle and lines) were produced in the normalized (x,y) coordinate space and converted to a PWM signal applied to each bimorph using Eqs 1–4. The micro-mirror focus power was set to 51% to obtain a focused spot, and the laser diode power was set to 100% to maximize the spot intensity on the paper screen.

**FIGURE 5:**
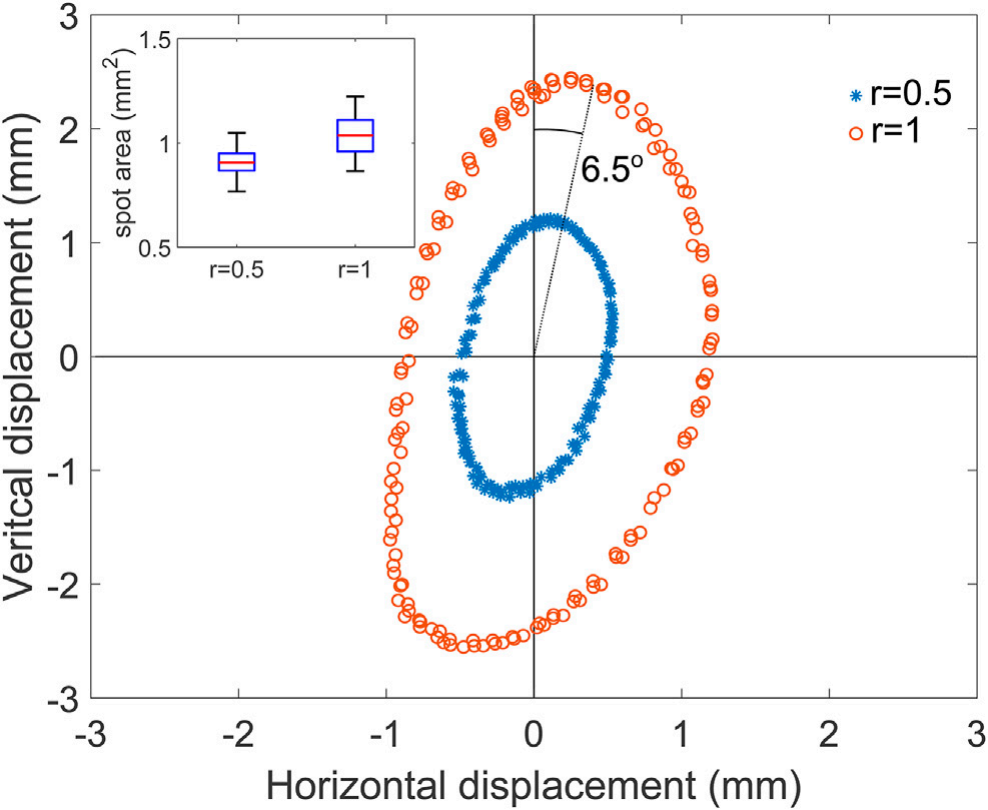
Measured laser spot position for a target circle of normalized radius of 0.5 (blue) and 1 (orange). Inset: box-plot of the spot area based on all the measured points.

First, a circular shape was performed at two normalized radii *r*: 0.5 and 1. This means that normalized setpoint coordinates x and y in Eqs 1–4 were obtained with *x* = *r* cos (*ωt*) and *y* = *r* sin (*ωt*). The period of the circle was set to 10 s, and images were recorded for 30 s at 5 Hz (limited by the camera exposure time). The movie was then processed frame by frame using the pipeline described before to obtain the spot position in real-world coordinates. An ellipse was fitted to the *r* = 0.5 test data to identify the center of the path, and all data points were then shifted to center the figure at the origin (Figure 5). The vertical and horizontal axes of the figure are the axes of the image and not those of the micro-mirror tilt axes. The user can set the rotation between the two frames of reference, as the PCB is mounted on a *x* − *y* − *θ* stage, which enables to freely choose the rotation between the camera (or cell stretcher) axis and micro-mirror axes. The micro-mirror used for this test clearly provides more displacement along one axis, transforming the intended circle into an ellipse. Table 1 gives the semi-axes length obtained by fitting an ellipse on the data point. It shows that due to one of the micro-mirror axes having a larger displacement, the ellipse is elongated by a factor of 2.5. The size of the ellipse is proportional to the applied power: the length of the semi-axes double when the applied power doubles and the elongation remains unchanged.

**TABLE 1:** Semi-axes and elongation of the ellipse fitted on the data from Figure 5.

	a (mm)	b (mm)	Elongation
r = 0.5	0.49	1.21	2.47
r = 1.0	1.01	2.50	2.48

**FIGURE 6:**
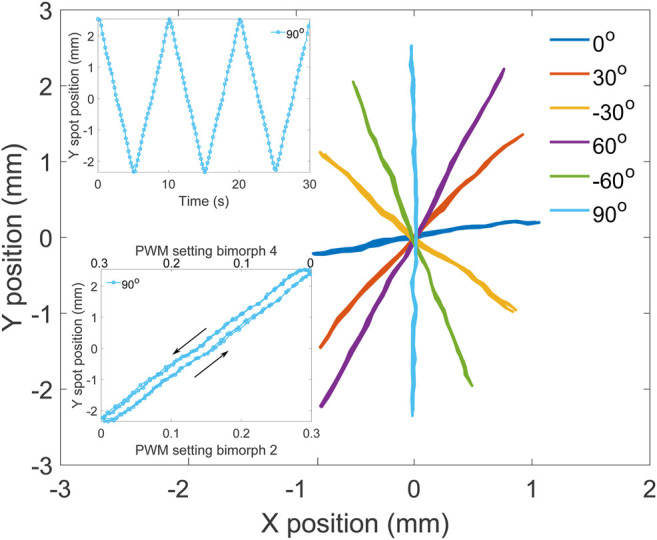
Measured laser spot position in real-world units for target lines with different orientations. Top inset: Y position of the spot for the 90° line as a function of time. Bottom inset: Y position of the spot for the 90° line as a function of the bimorph power for the two actuators (2 and 4) controlling the *y* axis.

To further characterize the steering of the light over the target screen, we traced lines with different orientations, keeping the same parameters as for the circle. The lines had a normalized length of 2 and slopes of 0°,±30°,±60°, and 90° (Figure 6). The spot moved back and forth along each line with a period of 10 s, and data were collected for three periods (top inset). The ellipse from the previous test (Figure 5) is tilted clockwise, showing that the camera and the micro-mirror frames of reference were rotated. To account for this, the processed data from the line test were rotated by 6.55° counterclockwise, to make the 90° line vertical, as shown on the figure. The path followed by the spot forms straight lines, and the points from the three periods are superposed, showing excellent reproducibility. The line at 0° uses bimorphs 1 and 3 exclusively, while the 90° lines relies on bimorphs 2 and 4 (c.f. Figure 4A). For this micro-mirror, the vertical axis is clearly working better and provides more tilt. In addition to the different amplitudes of the two micro-mirror axes, we also notice that their motion is not perfectly orthogonal. The data were rotated to align the 90° line with the vertical direction. However, after the rotation, the 0° line is not perfectly horizontal. But despite its imperfection, this micro-mirror enables to reach points within an ellipse with semi-axes of 1.01 and 2.50 mm (c.f. Table 1), i.e., an area of 7.93 mm^2^. The relation between the power applied to bimorphs 2 and 4 and the spot displacement on the screen is linear and present a small hysteresis, as shown for the 90° line (Figure 6 bottom inset).

**FIGURE 7:**
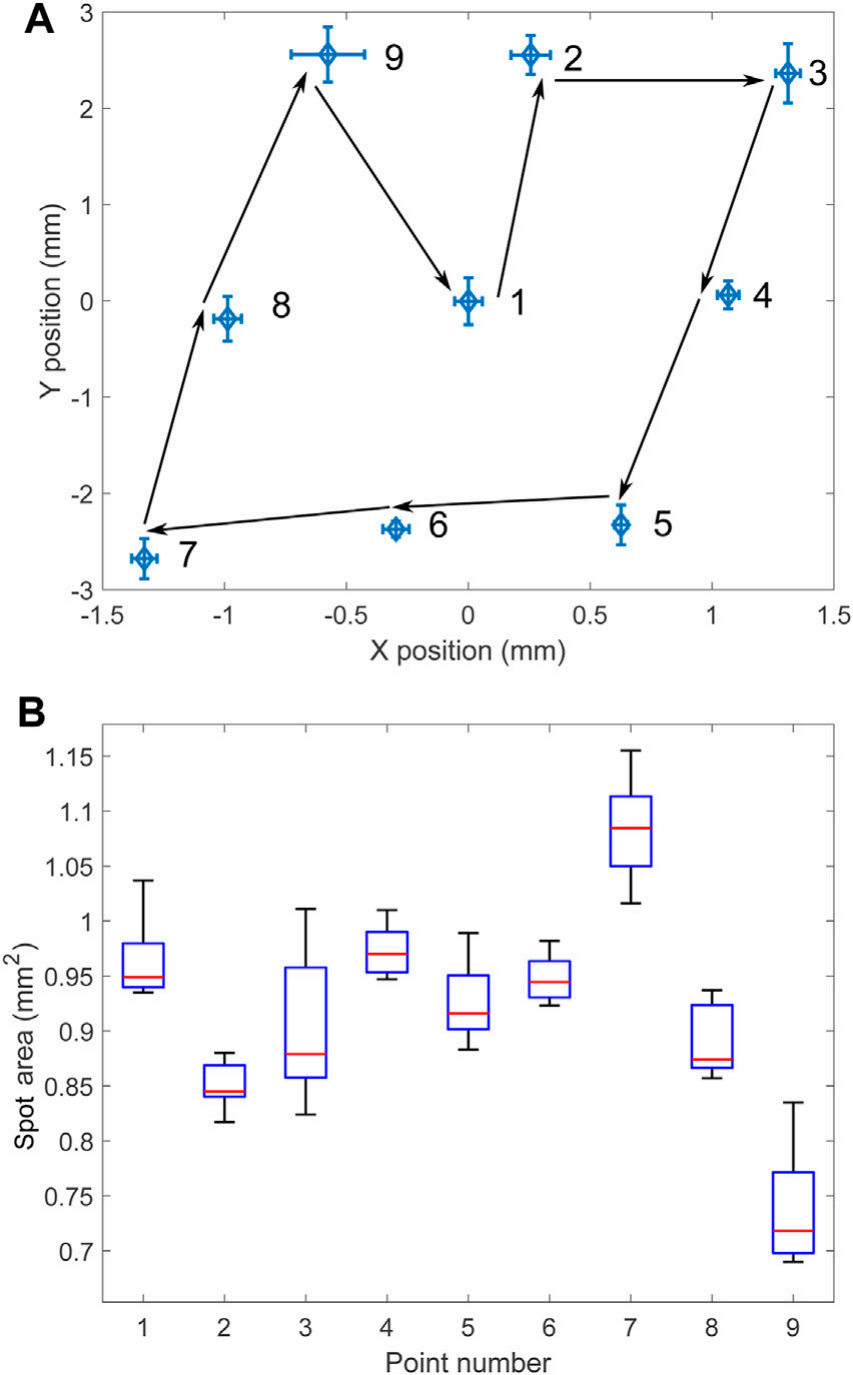
**(A)** Average spot position when jumping between 9 points in the sequence indicated by numbers and arrows for 10 cycles. The error bars indicate the standard deviation along both directions. The amplitude of the error bars is multiplied by 10. **(B)** Spot area at the different locations.

The reproducibility of the light positioning was tested by repeatedly jumping between the nine points with the x and y setpoint coordinates taking the possible permutations of the values −1, 0, and 1. All parameters were kept identical to the previous tests, and the pattern was repeated 10 times. Figure 7A shows the average spot location at the nine different positions. The positioning of the laser is very reproducible, with the error bars on the figure representing the standard deviation in both directions, magnified by a factor 10. Detailed values of the standard deviation are given in Table 2. Figure 7B shows a box plot of the laser spot area at the nine positions. The spot size (and shape) changes when the micro-mirror tilts, but this could be corrected by adjusting the micro-mirror power as a function of the micro-mirror tilt.

**TABLE 2:** Standard deviation *σ* in *x* and *y* in the location of the 9 points, calculated over 10 cycles.

Point	1	2	3	4	5	6	7	8	9
*σ*_*x*_ (µm)	5.7	8.1	5.0	4.4	2.7	5.4	5.2	5.7	15.1
*σ*_*y*_ (µm)	24.4	20.3	30.8	14.6	20.6	8.3	21.0	23.2	28.4

**FIGURE 8:**
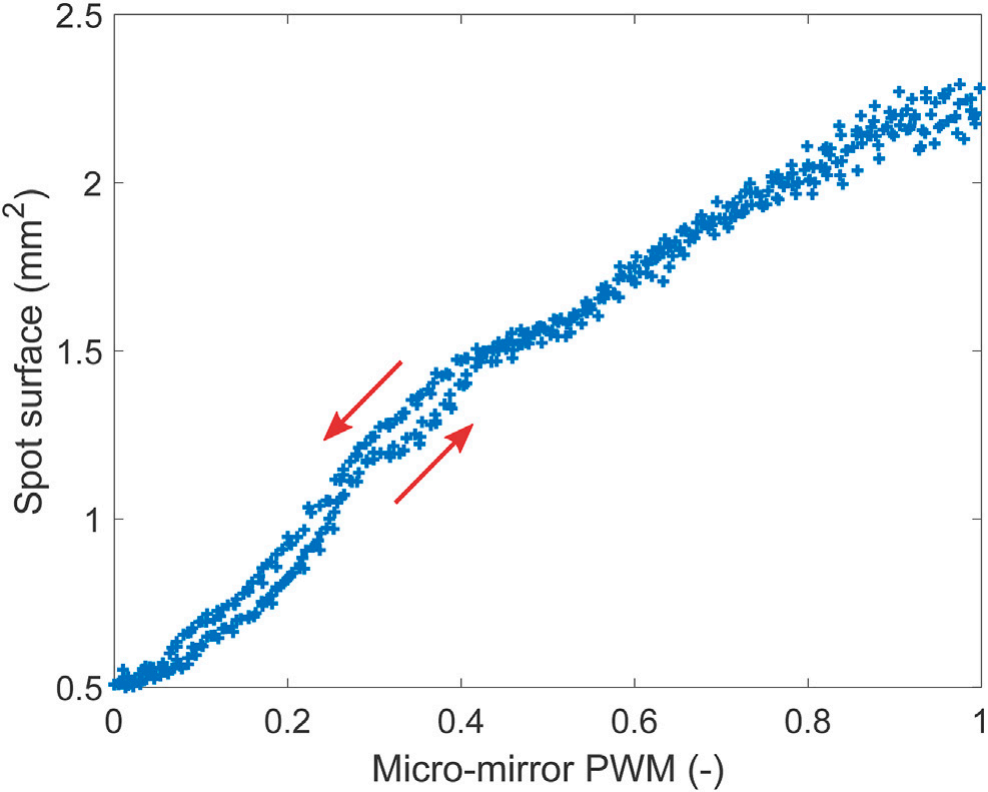
Spot diameter as a function of the heating power applied to the mirror. The higher the heating power, the higher the radius of curvature.

The ability to control the size of the laser spot by changing the micro-mirror curvature is shown in Figure 8. For this test, the distance between the micro-mirror and the optical fiber (as visible on Figure 3) was adjusted so that the spot was focused on the screen (i.e., smaller area) when no heating was applied to the micro-mirror, which was therefore in its maximal curvature configuration. The bimorphs were kept in the neutral (0,0) position, and the laser diode used at maximal intensity. The PWM signal controlling the heating of the micro-mirror was then ramped up and down between 0 and 1 with a period of 30 s, and data were acquired for four periods. This actuation signal caused the micro-mirror to open up and close, leading to a modulation of the focal length, and hence a change in the laser spot size on the screen was observed. The size of the spot was 0.5 mm^2^ when focused on the screen, and its area increases when the mirror flattens, up to a maximal surface of 2.3 mm^2^. The surface of the laser spot can therefore be modulated by a factor 4.6. At low heating power, a hysteresis is visible (Figure 3), and the arrows indicate the direction of the ramp. The hysteresis is hidden by noise at higher heating power, which is caused by the low intensity of the spot that makes it challenging for the image processing script to identify the edge of the spot correctly.

**FIGURE 9:**
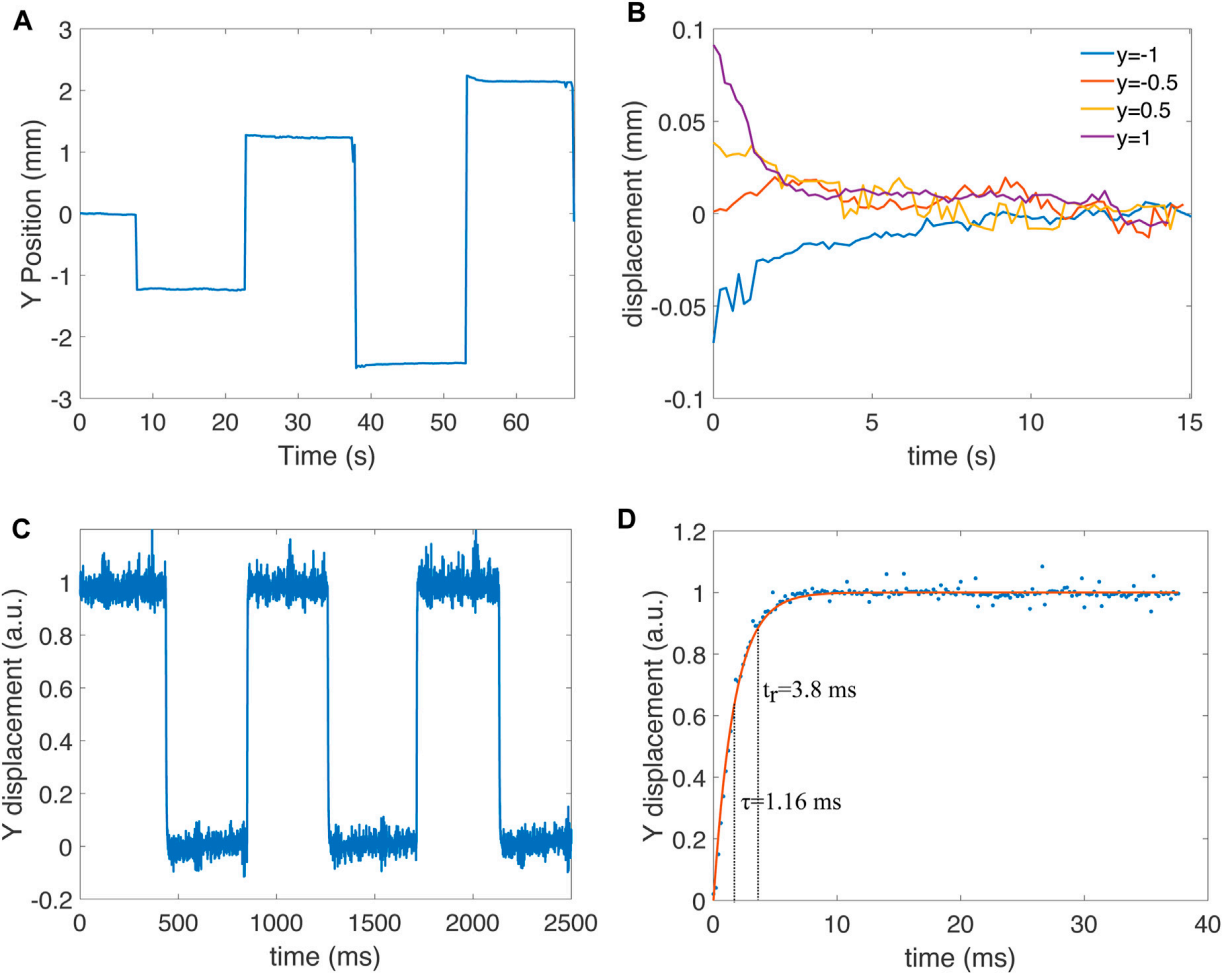
**(A)** Characterization of positioning stability by staying 15 s at normalized position *y* =−0.5, *y* =+0.5, *y* =−1 and *y* =+1. The initial position is at *y* =0, which is held a few seconds at the start of the measurement. **(B)** Holding plateau for the four jumps from **(A)** shown as a difference relative to the value at the end of the plateau to remove the offset. **(C)** Jump between normalized position *y* =0 and *y* =+1 measured with a photodiode with a sampling time of 200 µs. **(D)** Detailed view of a raising step from measurement **(C)** and exponential fit. The fit leads to a time constant of *τ* =1.16 ms and a rise time (time to reach 90% of the final value) *t*_*r*_ =3.8 ms.

Because thermal actuators are subject to time constants of different scales, we characterized the position of the spot when the bimorphs were held at a constant power setting for 15 s. Indeed, although the primary time constant of the bimorphs is in the order of milliseconds, long thermalization time constants may exist, caused by the heating of the micro-mirror itself or the base of the chip. This slow heating can lead to a drift of the spot position when power is applied continuously for several seconds. For this test, we only used the *y* axis of the micro-mirror, and kept the *x* axis at the baseline power. The *y* axis started at the normalized position 0, and then jumped to −0.5, +0.5, −1, and +1, staying 15 s at each value (Figure 9A). The spot position remains stable, although a small amplitude decrease is observed for the position of −1 and +1. For a better quantification of the long thermalization time constant, we plotted the four plateaus as the relative displacement with respect to the final stable position reached at the end of the holding time (Figure 9B). There is a visible increase of spot drift for the two higher power values, and more drift in the positive region of the *y* axis, which indicates that bimorph 2 is less stable than bimorph 4. Nevertheless, the drift in laser position is smaller than 0.1 mm.

The response speed is another important time-related property of the system. The camera used for the characterization only provides a few frames per second, which does not allow us to characterize how fast the micro-mirror responds to a step-change in position. We therefore replaced the screen and camera with a four-quadrant photodetector (Thorlab PDQ80 A). We oscillated the position of the micro-mirror between *y* = 0 and *y* = 1 at ≈1 Hz (axis x kept at the baseline) and recorded the position of the spot on the photodiode with a sampling period of 200 µs. The voltage output of the photodiode was then normalized on a 0 to 1 scale (Figure 9C). To characterize the rise time, we plotted the first 38 ms of one of the rising edges, and we fitted an exponential charging curve (Figure 9D). The exponential fit leads to a time constant of *τ* = 1.16 ms and a rise time (time required to reach 90% of the final value) of *t*_*r*_ = 3.8 ms.

### 3.3 Combining Light Steering and the Cell-Stretcher

**FIGURE 10:**
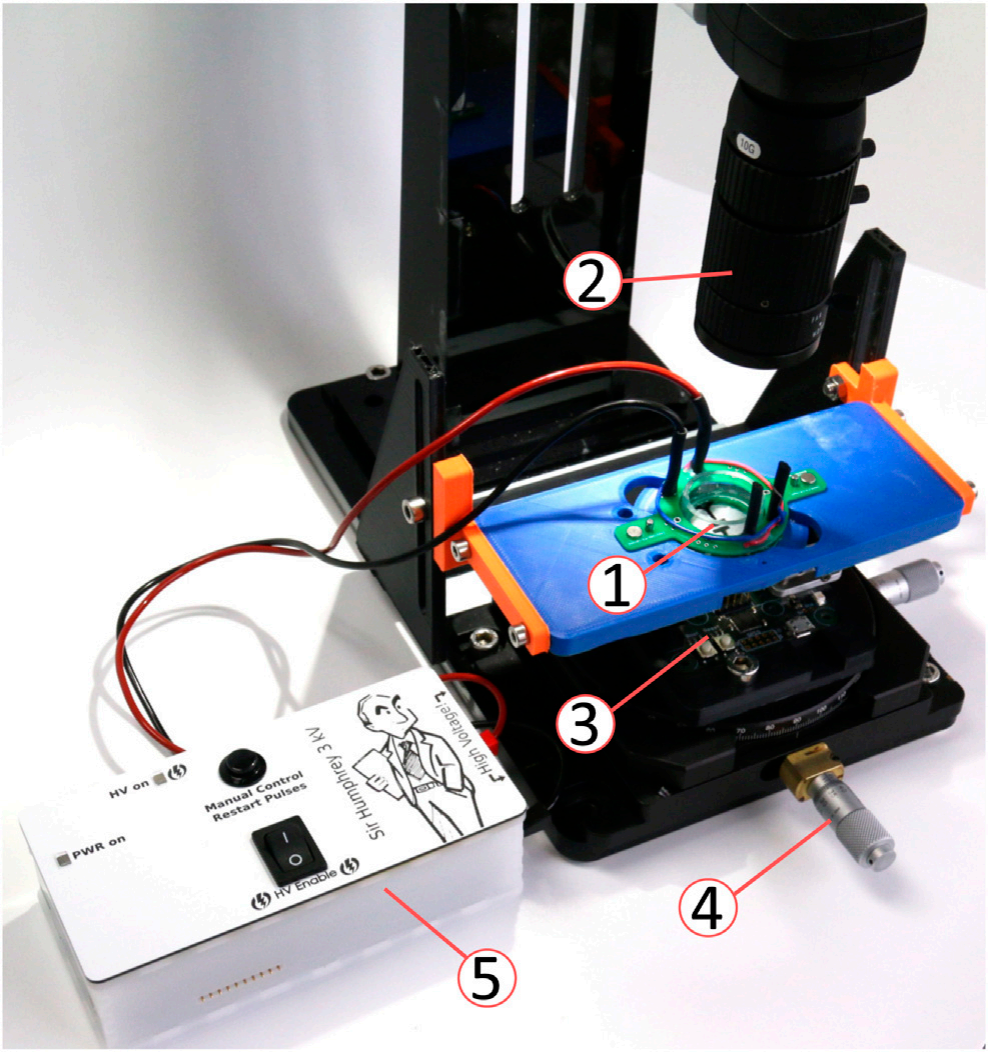
Experimental setup for the integration of the micro-mirror platform and the DEA cell stretcher. ➀ DEA cell stretcher with spring-loaded contact adapter, ➁ camera and objective, ➂ micro-mirror platform (c.f. Figure 4B), ➃ *x* − *y* − *θ* alignment stage, and ➄ Computer-controlled high-voltage power supply.

The platform with the micro-mirror was coupled with a cell-stretching device to demonstrate the ability to combine actuation with beam steering (Figure 10). A portable stand held a camera (IDS UI-3880LE-C-HQ) with a zoom lens (computar MLM-3XMP) and an *x* − *y* − *θ* stage. The stand provided a movable base that can be used in a sterile flow hood of a cell culture lab. The optogenetics platform (Figure 4B) was placed on the *x* − *y* − *θ* stage. A 3D printed holder with a magnetic base was used to hold the DEA cell-stretching device. A PCB with magnets and spring-loaded contacts provided electrical contact between the cell-stretcher and a computer-controlled high-voltage power supply. The cell stretcher holder could be moved to place the region of interest in the camera field of view. The stage holding the optogenetics platform could be moved independently of the cell-stretcher, thus making it possible to position the laser spot in its neutral position anywhere on the membrane surface. For these tests, no cells were cultured on the cell stretcher, but we have demonstrated the ability to stretch cells with DEAs of a similar design in earlier publications (Poulin et al., 2016; Imboden et al., 2019; Wu et al., 2022). The membrane was sprayed with barium titanate powder to visualize the laser spot. When cells are cultured on the device, the culture well is filled with cell culture media. However, this does not affect the control of the laser spot, as the laser is coming from the underside of the membrane. The micro-mirror used for this experiment was from a different batch than the one used for the characterizations of the previous sections. In particular, it has a lower initial mirror curvature of the mirror that prevents fully focusing the spot on the stretcher membrane. With this mirror, we set the maximal power applied to the bimorphs to *PWM*_max_ = 20%.

**FIGURE 11:**
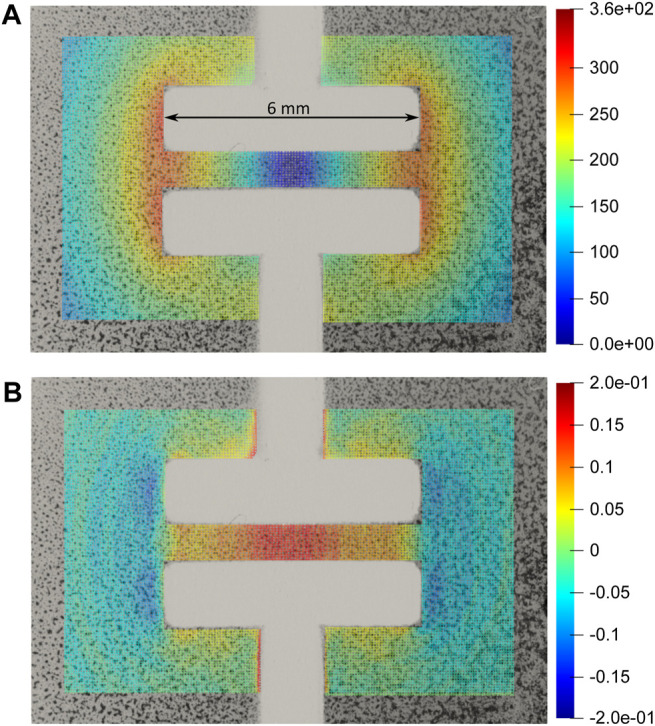
**(A)** Magnitude of displacement in µm with 2,400 V applied to the cell-stretcher. **(B)** Strain in the horizontal (*x*) direction at the same applied voltage. The culture area located between the electrodes generates strains between 5 and 20% depending on location, while the zones located outside of the active region see a slight compressive strain. The laser spot can address zones submitted to different strain amplitudes.

First, we characterized the actuation of the cell-stretcher for voltages up to 2,400 V. The average strain over the active area was calculated by measuring the length and width of the culture zone with and without actuation. The strain in the stretching (horizontal direction) reached 10.4%, while the strain in the perpendicular direction was −0.6%, that is, slightly compressive. The barium titanate sprayed on the membranes creates a random pattern that was used to generate a displacement map of the zone of interest using a subpixel phase–based image registration algorithm (HajiRassouliha et al., 2018). Figure 11A shows the magnitude of the displacement field for the maximal applied voltage (2,400 V). The maximal displacement is observed at the left and right edges of the electrode and reaches 360 µm. Figure 11B shows the strain field in the stretching (horizontal) direction. It shows that the strain is not homogeneous in the stretched area, but it peaks at 20% at the center of the device, and decreases to 5% on the sides.

**FIGURE 12:**
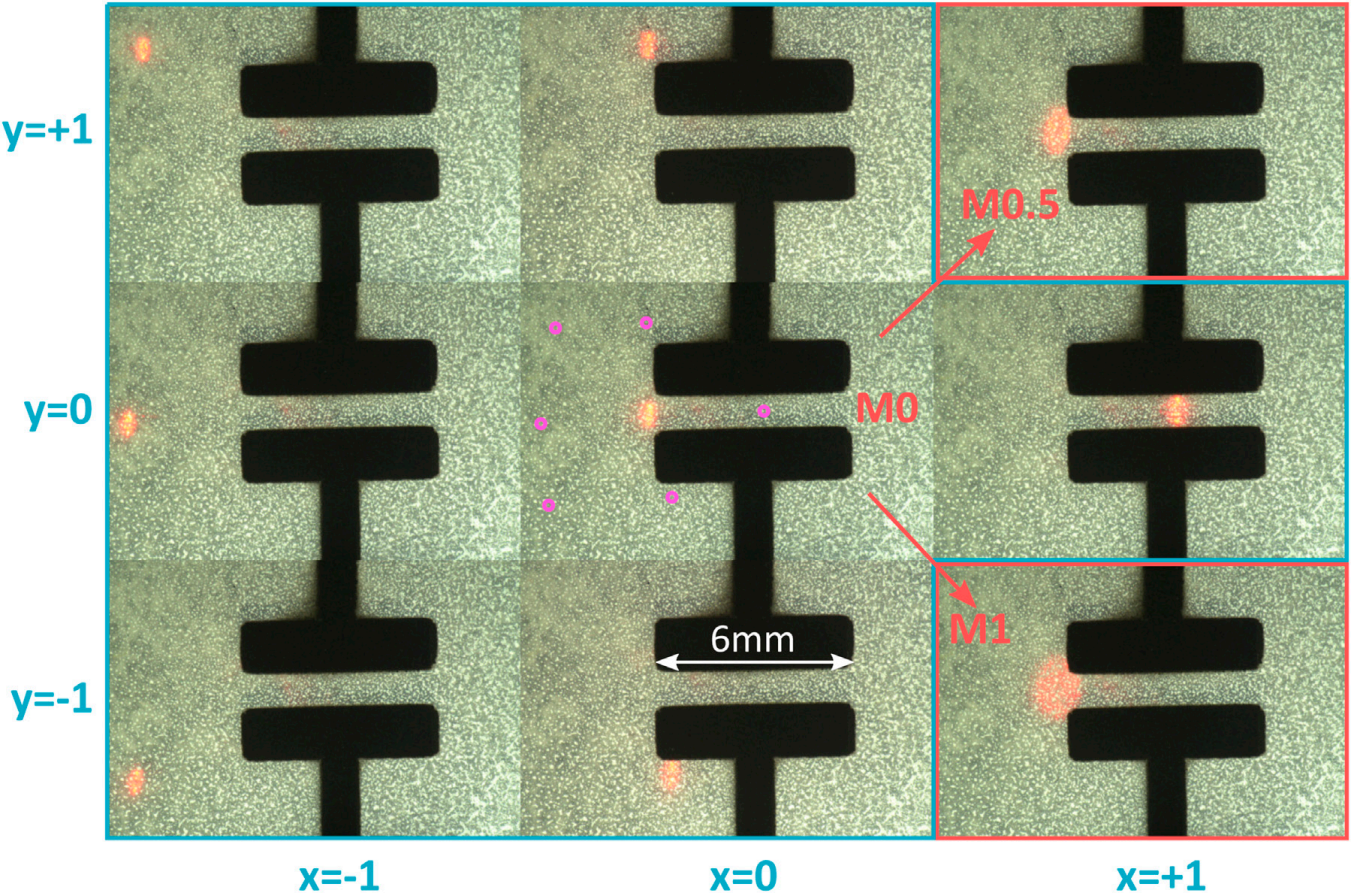
Laser spot moved over the cell stretcher membrane. The central image represents the neutral position, and the other images show the displacement of the spot along axes *x* and *y* in the relative coordinate system. All images are obtained with the mirror power at 0%, except for the top right and bottom right images, which show the spot in its neutral position with mirror power of 50 and 100%. The purple dots on the central picture indicate the laser spot position of the other pictures. The mirror enables to cover a zone of 6.9,×,5.7 mm^2^.

Figure 12 shows how the laser spot can be controlled over the cell stretcher membrane for a PWM signal limited to *PWM*_max_ = 20%. The *x* − *y* − *θ* stage is adjusted to place the laser spot on the edge of the electrodes. Because of the low initial curvature of this batch of mirrors, the most focused spot is achieved with no mirror heating power. The spot is then moved to the extremes of the relative coordinate systems. The bottom right and top right positions are not shown, as hidden below the electrodes. Instead, the central position with mirror heating power of 50 and 100% are shown. The purple circles on the central picture indicate the location of the laser spot from the other pictures. With this setting of a maximal PWM power (*PWM*_max_ = 20%), the laser spot can be controlled within a rectangular area of 6.9, ×, 5.7 mm^2^. This is large enough to address cells in the stretched area (i.e., between the electrode, see Figure 2C), and in the passive unstretched area. It also enables to target cells exposed to different peak strains values (c.f., Figure 11B).

In addition to addressing specific areas of the culture well, it is also important to be able to track cells while they are being stretched. For example, an application may require illuminating a group of cyclically stretched cells. Unless the group of cells is located at the center of the device, the membrane strain will also induce a translation. The micro-mirror enables direct control of the laser beam position, which can be steered to follow the cells of interest. To demonstrate this ability, we have written a LabVIEW program to synchronize the control of the micro-mirror with the actuation of the cell stretcher through a computer-controlled Peta-pico-Voltron high voltage power supply (Schlatter et al., 2018). For the test, the spot was located at the edge of the electrodes, in a similar position as the neutral position of Figure 12. This is the location of the stretcher that sees the largest translation: a 10% global strain of the electrodes in the horizontal direction leads to a translation of the left edge of the electrode by 300 µm. The program user interface displays a live image of the cell stretcher membrane. In a first phase, the user can apply a voltage to the stretcher and control the laser spot. A button can be pressed to add the current settings (actuation voltage and bimorph power) to a list of set points. Once the points have been defined, the second phase of the program can start in which the parameters saved previously are applied to the high-voltage power supply and the micro-mirror. At the same time, the camera films the actuation and laser motion. In a first test, we defined a 6-point ramp between 0 and 10% strain and back to 0, with the laser spot following the edge of the electrode. In a second test, we performed step jumps from 0 to 10% strain and followed the same point with the laser spot. The actuation voltage required to reach 10% strain on the actuator was 2,600 V. The video of the two tests is provided with the article’s supplementary information and demonstrates the ability of the laser spot to be synchronized with the actuation of the cell-stretcher.

## 4 Discussion

One of the challenges that still need to be addressed is the variability between batches of micro-mirrors due to process variation, different annealing treatments, etc. However, this can be addressed by implementing a device-specific calibration. When a new mirror is installed on the platform, a series of random bimorph power values between 0 and *PWM*_max_ can be generated. For each of these combinations, the user can click on the camera feed to identify the position of the spot in the image coordinate system. The process can even be automated by using image processing algorithm to identify the position of the laser on the image. Most of the data can then be used to train a machine-learning algorithm, while a small portion is kept to assess the model’s accuracy. This method would make it possible to adapt to a mirror replacement. In addition, it could also account for the aging of the mirror or changes in external parameters (temperature, etc.), as this calibration can be repeated when needed. There are also process variations in the fabrication of the DEA cell-stretchers that lead to different voltage–strain behaviors between devices. It includes differences in the thickness of the elastomer membrane or of the compliant electrodes. Culturing cells on the stretchers usually requires coating the silicone membrane with a protein layer such as collagen or fibronectin to promote cell adhesion, and the thickness of this coating is difficult to control and influences the actuation behavior. We have implemented an automated calibration routine for the cell stretcher, using image auto-correlation to track the corners of the electrodes. A voltage ramp is applied to the stretcher, and the electrode expansion measured to calibrate the cell-stretcher prior to an experiment.

There are cases when this open-loop approach might not be sufficient. For example, we have shown in Figures 9A,B that when the mirror is kept at the same position, the spot position can drift. This is especially visible if the bimorphs are actuated close to their maximal power, that is, when the spot approaches the edges of the addressable zone. For applications that require remaining at the same position for an extended period, and for which positioning accuracy is essential, a close-loop control mode can be implemented, using the camera feed and image analysis to locate the spot and actively control the bimorphs to keep the spot at the correct solution.

Working with the optogenetics platform involves two different size scales: the cell stretcher and laser spot tracking require a field of view of a few tens mm^2^, while cell observation requires magnifications about two orders of magnitude higher. Cell observation is usually performed using inverted microscopes, high-quality liquid-immersion objectives, and phase contrast. In its present state, our optogenetics platform does not allow the operator to observe the cells. However, it is designed for easy installation and removal of the cell stretcher, thanks to the magnetic holder. The cell-stretcher typically needs to be kept for several days in an incubator until the cell culture is confluent. Confluence and cell health can easily be checked on a high-resolution microscope before placing the device on the platform. Once on the platform, one can select the areas that need to be illuminated and perform the desired experiments. The laser spot can be focused down to a surface of 0.5 mm^2^ (Figure 8). It can therefore illuminate groups of cells in the stretched and unstretched zone, but it does not permit the illumination of single cells. The maximal intensity of the laser used on our platform is 1 mW, leading to intensities up to 2 mW mm^−2^, corresponding to the range of intensities used in the first optogenetic demonstration of light-induced neuron firing (Boyden et al., 2005). We have monitored the temperature of the well while illuminating the stretcher with media in the culture well and did not record an increase in temperature due to the illumination. The wavelength of the laser used in this study (635 nm) enables to activate red-shifted opsins such as red-activatable channelrhodopsins (ReaChR) (Lin et al., 2013), but the platform can easily accommodate different laser diodes to match the wavelength required to activate the relevant opsins (typically from 470 to 630 nm). For example, Channelrhodopsin-2 (ChR2) that enables the activation of neuronal spiking requires an excitation wavelength of 470 nm (blue) (Boyden et al., 2005).

Now that we have demonstrated the platform’s ability to steer a laser beam of variable size over a mechanically active cell culture membrane and synchronize the mirror actuation with the expansion of the soft actuator, the next stage will consist in using the platform for optogenetics experiments. In a previous work, we have already demonstrated different cell culture protocols on DEA-based soft stretchers (Poulin et al., 2016; Imboden et al., 2019; Wu et al., 2022). Supplementary Figure S1 shows rat cardiomyocytes patterned on the cell-stretcher. For this application, an action potential was triggered using electrical stimulation and traveled along the patterned strand of cardiomyocytes, which could be rapidly stretched by the soft actuators (Imboden et al., 2019). However, electrical stimulation has limitations, including difficulty changing the targeted area and toxicity to the tissue or lack of cellular specificity (Ferenczi et al., 2019). The next stage will consist in replacing the electrical stimulation with optical stimulation. This requires transfecting cells with light-sensitive opsins (e.g., rhodopsin) and will be the focus of our next study where we will use the platform for biological experiments.

## 5 Conclusion

We have demonstrated the integration of a steerable micro-mirror and a cell-stretching device capable of stretching cells into an integrated platform that combines optical and mechanical stimulation of cells. The cell stretcher based on soft actuators offers a compact solution to strain cells on a soft membrane. The transparency of the membrane enables the cell stretcher to be combined with the steerable micro-mirror, thus forming a platform unlocking new experiments bridging mechanotransduction and localized light activation. For optogenetics applications, cells are genetically modified so that specific pathways can be gated using light of a defined wavelength. They can then be plated on the cell stretcher and selective optical addressing enables to trigger or silence these pathways. Selective light illumination can also be used with light-activated drugs, making it possible to activate a therapeutic agent in a defined area of the cell culture. Our current platform can position a laser spot of variable size in a region an area of several square millimeters (40 mm^2^ for the mirror used for the tests with the cell stretcher, shown in Figure 12). The laser spot position and size, the light intensity of the laser diode, and the actuation voltage applied to the cell stretcher can easily be controlled from a computer *via* RS-232 serial communication. The DEA cell stretcher is made using a silicone membrane with low viscous losses and can therefore actuate to 10% strain in less than 1 ms. This contrasts with pneumatic cell stretchers, whose bandwidth is limited to a few Hertz. Similarly, the micro-mirror has a time-constant of 1.16 ms and can change position on the same timescale as the cell stretcher. This makes it possible to target applications with strain rates beyond physiological limits and study trauma such as commotio cordis or traumatic brain injury.

## Data Availability

The raw data supporting the conclusion of this article will be made available by the authors, without undue reservation.

## Footnotes

1 Tension Cell Stretching Bioreactor System, https://www.flexcellint.com/category/tension

2 Automated Cell Stretching System STB-1400, https://strexcell.com/cell-stretching-system/#STB-1400
