# Supplementary material for: The Integration of Optical Stimulation in a Mechanically Dynamic Cell Culture Substrate
Source: Front Bioeng Biotechnol. 2022 Jul 19;10:934756. doi: 10.3389/fbioe.2022.934756 (PMC9344002; doi:10.3389/fbioe.2022.934756)
Supplement: Supplementary file 1 [file DataSheet1.PDF]

Supplementary Material

1 SUPPLEMENTARY VIDEO

The supplementary video demonstrates the motion of the bimorphs and mirror focus using PWM signals, the control of the spot location on the membrane of a cell-stretcher using a joystick, and the synchronisation between cell-stretcher actuation and laser spot tracking.

2 CELLS ON CELL-STRETCHING DEVICE

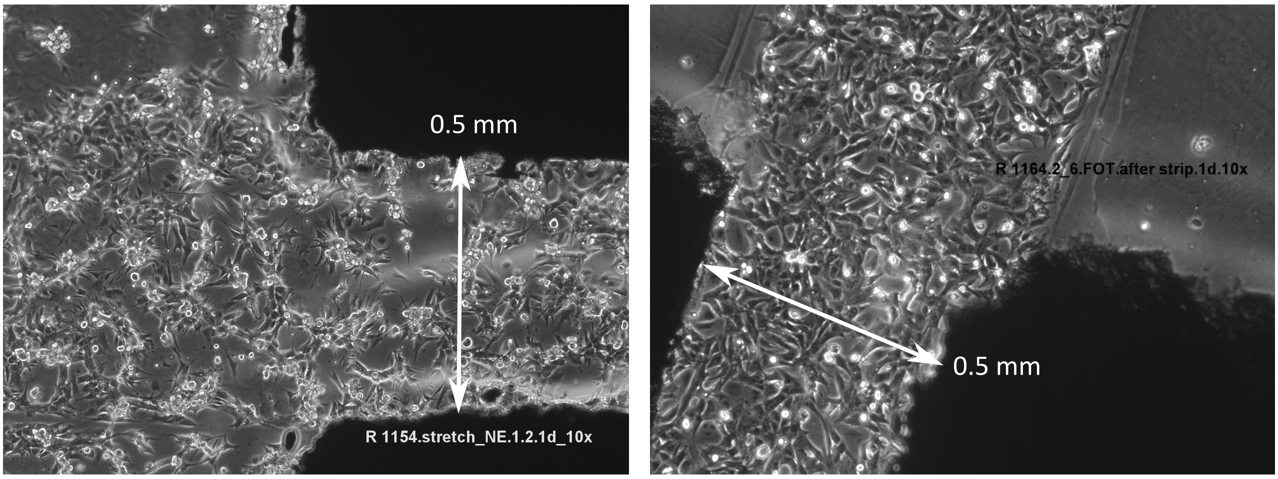

Figure S1. Neonatal rat ventricular cardiomyocytes patterned using a lift-off technique with a Mylar mask on the dielectric elastomer actuators (Imboden et al., 2019). Prior to cell plating, the actuator membrane was coated with collagen type IV to promote cell adhesion on the silicone membrane.

3 OPTOGENETICS MEMS CONTROLLER

Figure S2 shows a 3D rendering of the printed circuit board (PCB) designed to control the micromirror. The main components are detailed in Figure 4b of the article. The circuit consists of 3 main parts (Figure

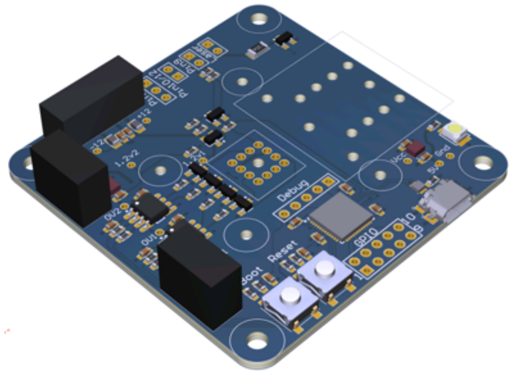

Figure S2. Printed Circuit board of the optogenetics MEMS controller

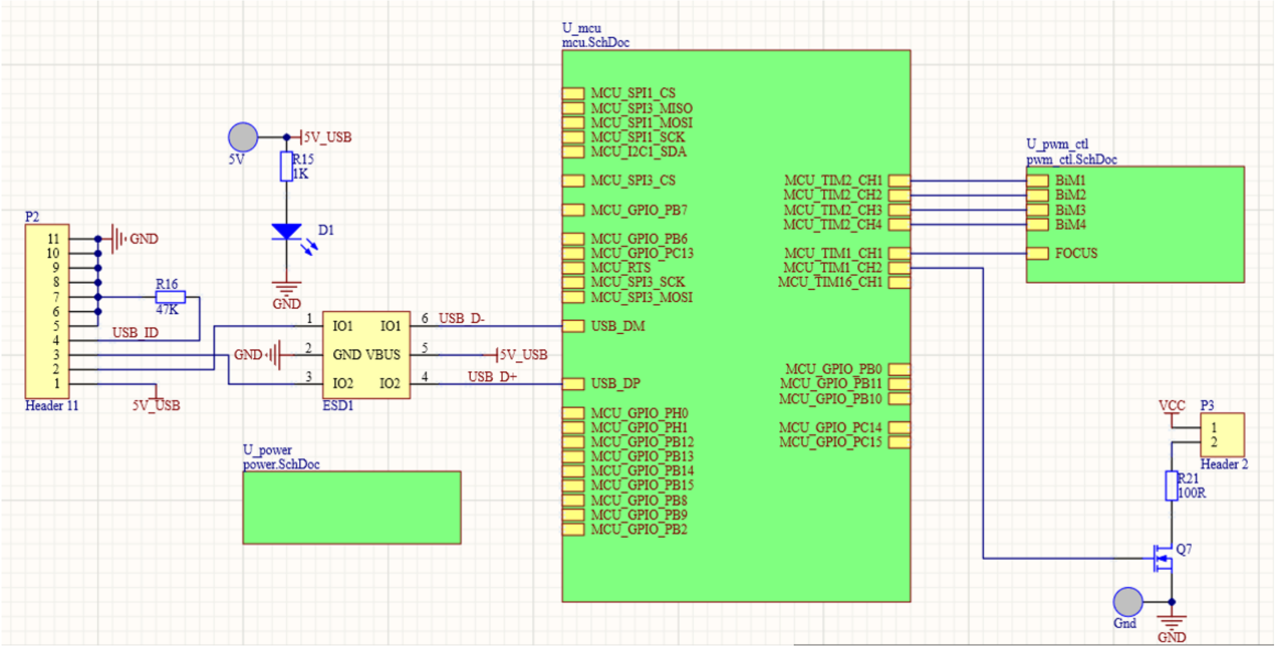

Figure S3. Main component of the circuit schematics. The header P3 controls the laser diode.

S3). A USB connector is used to power the platform and to communicate with the microcontroller. A power management block transforms the USB's 5 V into the various voltage levels required to operate the bimorphs and the other components. The microcontroller (STM32L433) listens to commands sent on the USB bus and generates 6 PWM signals (4 bimorphs, the micro-mirror focus, and the laser diode intensity).

Figure S4 describes the power management block. DC/DC converters are used to create the various voltage levels required to drive the circuit. 2 sets of floating voltages (3.3 V and 1.2 V) are generated to control the bimorphs. A 3.3 V voltage referenced to ground (VCC) powers the microcontroller, the active ICs, and the digital inputs (Boot and reset switches). A ±12 V DC/DC converter generates the voltage required to control the micro-mirror's focus.

Control of the bimorphs and micro-mirror focus is shown in Figure S5. First, the PWM signals for the 4 bimorphs are shifted to the two floating voltage levels using high-speed CMOS digital isolators (U1 and U2). A voltage of 1.2 V is applied to each bimorph through NMOS transistors (Q3-Q5) controlled by their respective PWM signals. One side of bimorphs 3 and 4 is biased at -12 V, while the opposite sides of bimorphs 1 and 2 are connected to 12 V via transistors Q1-Q2. These transistors are controlled by the focus PWM signal and modulate the heating power. When Q2 is off, resistors R25 and R26 bias bimorphs 1 and 2 to -12 V, i.e. at the same voltage as bimorphs 3 and 4. There is no current flowing through the mirror's centre in this situation. The PWM focus signal enables one to increase the voltage bias of bimorphs 1 and 2 with a difference of up to 24 V with respect to bimorphs 3 and 4, thus controlling the current flowing through the centre of the mirror. Because the resistance of the four springs holding the mirror (10s of kΩ) is much larger than that of the bimorphs (a few Ω), the mirror heating current is low and creates most of the Joule heating in the mirror area, and not much in the bimorphs.

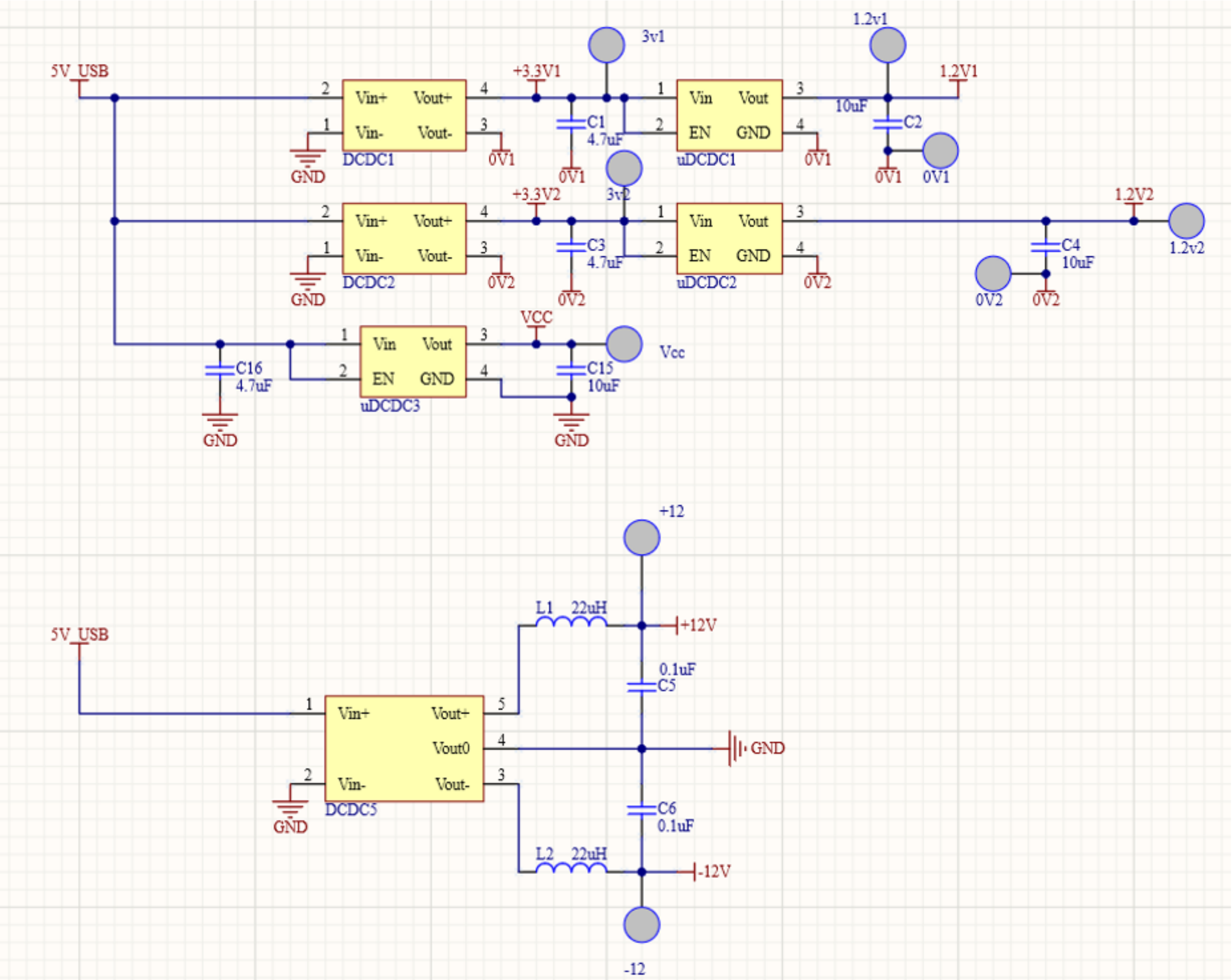

Figure S4. Power management.

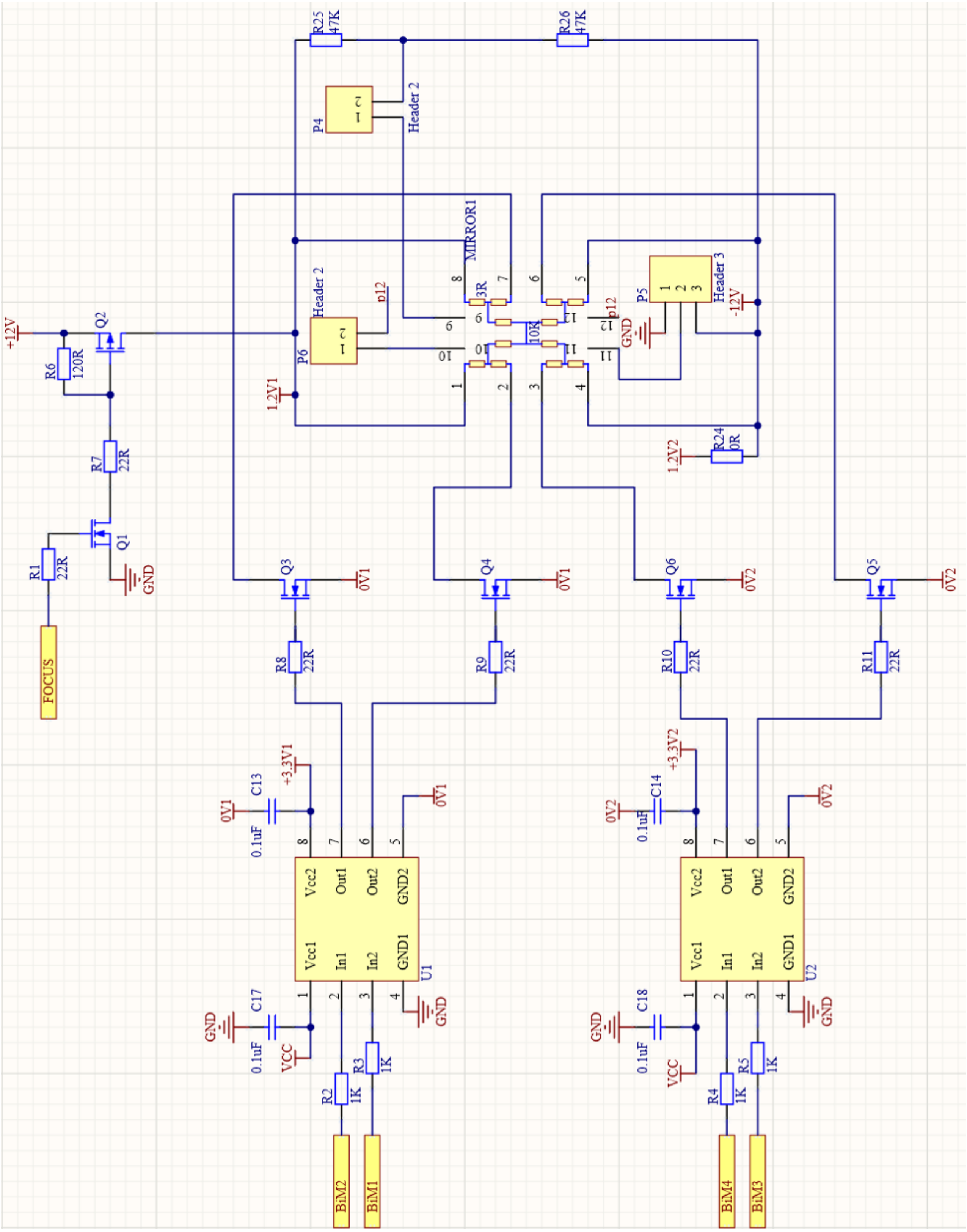

Figure S5. Bimorphs and micro-mirror focus control.

REFERENCES

Imboden, M., de Coulon, E., Poulin, A., Dellenbach, C., Rosset, S., Shea, H., et al. (2019). High-speed mechano-active multielectrode array for investigating rapid stretch effects on cardiac tissue. *Nature Communications* 10, 834. doi:10.1038/s41467-019-08757-2
